# Anomalous salting-out, self-association and p*K*_a_ effects in the practically-insoluble bromothymol blue

**DOI:** 10.5599/admet.1822

**Published:** 2023-05-23

**Authors:** Alex Avdeef

**Affiliations:** in-ADME Research, New York, NY 10128 USA

**Keywords:** salting-out effect, self-interaction, aggregation, solubility-pH, intrinsic solubility, ionization constant, dimerization constant

## Abstract

**Background and Purpose:**

The widely-used and practically insoluble diprotic acidic dye, bromothymol blue (BTB), is a neutral molecule in strongly acidic aqueous solutions. The Schill (1964) extensive solubility-pH measurement of bromothymol blue in 0.1 and 1.0 M NaCl solutions, with pH adjusted with HCl from 0.0 to 5.4, featured several unusual findings. The data suggest that the difference in solubility of the neutral-form molecule in 1M NaCl is more than 0.7 log unit lower than the solubility in pure water. This could be considered as uncharacteristically high for a salting-out effect. Also, the study reported two apparent values of p*K*_a1_, 1.48 and 1.00, in 0.1 M and 1.0 M NaCl solutions, respectively. The only other measured value found for pK_a1_ in the literature is -0.66 (Gupta and Cadwallader, 1968).

**Experimental Approach:**

It was reasoned that the there can be only a single p*K*_a1_ for BTB. Also, it was hypothesized that salting-out alone might not account for such a large difference in solubility observed at the two levels of salt. A generalized mass action approach incorporating activity corrections for charged species using the Stokes-Robinson hydration equation and for neutral species using the Setschenow equation, was selected to analyze the Schill solubility-pH data to seek a rationalization of these unusual results.

**Key Results:**

BTB reveals complex speciation chemistry in saturated aqueous solutions which had been poorly understood for many years. The appearance of two different values of pK_a1_ at different levels of NaCl and the anomalously high value of the empirical salting-out constant could be rationalized to normal values by invoking the formation of a very stable neutral dimer (log *K*_2_ = 10.0 ± 0.1 M^-1^). A ‘normal’ salting-out constant, 0.25 M^-1^ was then derived. It was also possible to estimate the ‘self-interaction’ constant. The data analysis in the present study critically depended on the p*K*_a1_ = -0.66 reported by Gupta and Cadwallader.

**Conclusion:**

A more reasonable salting-out constant and a consistent single value for p*K*_a1_ have been determined by considering a self-interacting (aggregation) model involving an uncharged form of the molecule, which is likely a zwitterion, as suggested by literature spectrophotometric studies.

## Introduction

The intrinsic (*i.e.*, neutral form) solubility of an ionizable substance in water generally *decreases* when a ‘water-structure maker’ salt (*e.g.*, NaCl) is added to the solution. The phenomenon is known as ‘salting-out’ [1-5]. The decrease in solubility seldom exceeds 0.3 log unit per molar salt. Such changes in magnitude are comparable to those seen between different polymorphs of a drug substance [6] and in solubility differences between racemates and individual isomers of an optically-active drug substance [7-9].

In a simple view of the salting-out phenomenon, ions introduced from the salt (*e.g*., Na^+^, Cl^–^) become strongly hydrated, thus tying up a significant portion of the available water. The added neutral organic solute, effectively deprived of the water molecules associated with the salt ions, dissolves to a lesser extent in terms of the total solution volume, thus appearing to be less soluble.

Many studies of the salting effect had focused on relatively simple organic nonelectrolytes, including gases and water-immiscible organic solvents [2-4,10-12]. The more soluble the nonelectrolyte, the larger is the salting effect [11]. However, there appear to be very few reported salting studies of practically-insoluble (*e.g*. intrinsic solubility, *S*_0_ < 10^-5^ M) nonelectrolytes, particularly of drug-like molecules, or substances of similar complexity.

**Figure 1.**
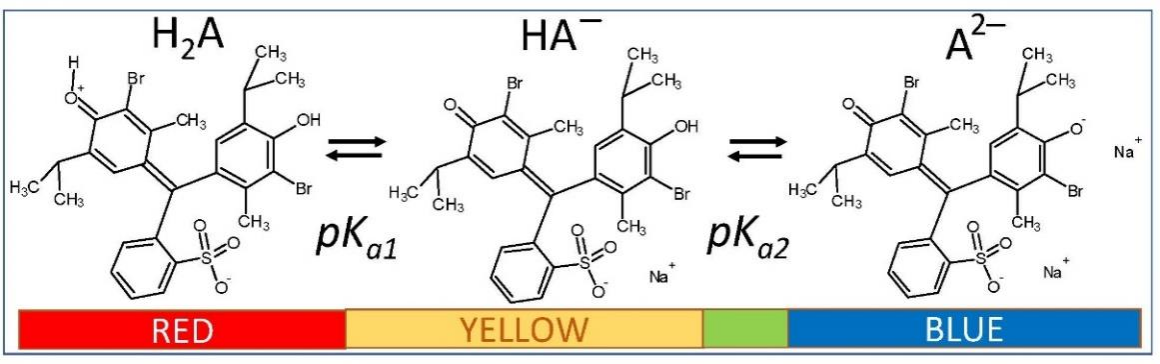
Ionization equilibria for the diprotic weak acid bromothymol blue in water. Although the value of p*K*_a2_ is confidently established, the value of p*K*_a1_ has been elusive.

In this communication, we re-examine the saturation solution-pH behavior of bromothymol blue (BTB, Fig. 1), which is practically insoluble in its zero net-charge form in strongly acidic solutions (left-most structure in Fig. 1). The molecule may be used as an indicator (yellow for pH <6, blue for pH >7.6, and green in between) to follow pH-dependent cellular processes. It can function as a complexing agent, and has been used in textiles, paints, cleaning products, detergents, and photovoltaic cells [13]. In cases of prolonged general exposure or if absorbed through the skin, BTB can be harmful. Because of its low solubility and high stability, BTB can be a persistent environmental pollutant [13].

BTB is a diprotic acid (H_2_A), with ionization reactions shown in Figure 1. In strongly acidic solution (pH <1), the molecule is plausibly a zwitterion forming a red solution. In mildly acidic/neutral and alkaline solutions, BTB exists as the anionic species HA^–^ and A^2–^. The second ionization constant (p*K*_a2_) has the reported value of 7.12 at ionic strength, *I* = 0.1 M [14]. In contrast, the value of p*K*_a1_ has been unresolved for many years, with values reported as low as -0.66 [15] and as high as 1.48 [14]. There has also been a long-standing controversy about the structure of the uncharged species in aqueous solution (pH <1).

**Figure 2.**
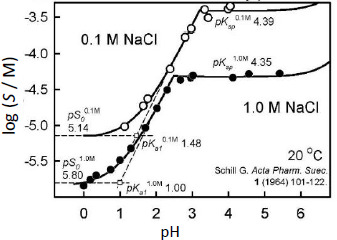
Bromothymol blue solubility-pH profiles reported by Schill [14], with data collected at 20 °C, after 24 h equilibration time. The upper saturated solutions profile contained 0.1 M NaCl, while the lower profile contained 1.0 M NaCl. The pH electrode was calibrated by Schill for the *concentration* scale, using solutions of known [H^+^] concentrations. The various listed equilibrium constants are those reported by Schill.

Schill [14] extensively studied the solubility-pH behavior of BTB in 0.1 M and 1.0 M NaCl solutions at 20 °C. In a series of publications, Schill applied BTB as a sensitive ion-pair extraction reagent to determine the concentrations of amines and quaternary ammonium compounds by spectrophotometry. The reported BTB solubility-pH data, along with the originally-determined constants are shown in Figure 2. We found three points of interest in Schill’s study, which appeared in need of additional examination.

- The study indicated surprising departures from the expected salting behavior of neutral electrolytes. The difference between the apparent intrinsic solubility in pure water and in solutions containing 1.0 M NaCl was reported to be greater than 0.7 log unit for BTB, which is inexplicably high [1-5].
- Also, two significantly different values of the first ionization constant were reported by Schill in saturated solutions: p*K*_a1_ = 1.48 in 0.1 M NaCl and p*K*_a1_ = 1.00 in 1.0 M NaCl (*cf*. Fig. 2). The Stokes-Robinson hydration equation [16] (a) includes the Debye-Hückel expression (ion-ion electrostatic interactions), (b) incorporates decrease in activity of water (work done in immobilizing some of the bulk water to hydrate ions), and (c) accounts for the free energy change of ions (as their concentrations increase when the volume of bulk water decreases upon hydration of ions). From the Stokes-Robinson hydration equation, the difference between the apparent p*K*_a1_ values is expected to be near 0.02, far less than the above 0.48 implied value [14].
- Schill’s examination of the UV spectra of BTB in sub-saturated solutions suggested that (AH^–^)_2_ and (AH^–^)_4_ aggregates might be forming in the pH region where the BTB anion prevails. They reported association constants from analysis of the spectroscopic data in the absence of visible signs of precipitation. In saturated solutions in the same region, micelles appeared to form. The reported limiting slope in Figure 2 in the pH 1-3 region is +1, which is not compatible with the aggregation models proposed by Schill. If aggregation comprised solely of the HA^–^ species, the slopes in the acid region would have been +2 (dimer) or +4 (tetramer), rather than +1 [17]. If mixed-charge aggregates (H_2_A.HA^–^)_n_ were to form, the slope would be +1 [17], but such models cannot resolve the unusual salting-out magnitude reported by Schill. So, the stoichiometry of the observed aggregation may need to be further examined.

In the present communication, an attempt is made to rationalize the apparently excessive salting behavior, the lack of consistency regarding the value of p*K*_a1_, and the formation of aggregates by applying a mass action model, using the program *p*DISOL-X™ (*in-ADME* Research) [16-26], to analyze the saturation solubility-pH data published by Schill [14]. It was hypothesized that the critical aggregation, which could explain some of the above-mentioned anomalies in acidic solutions (pH <3), is due to the self-association of the *zero net-charge* species, H_2_A.

## Method

### Mass action equilibrium model

Consider a diprotic acid, H_2_A, with the dissociation constants p*K*_a1_ and p*K*_a2_. Let’s assume it is prone to form zero net-charge dimers, (H_2_A)_2_, in aqueous solutions. The equilibrium reactions of relevance in saturated solutions (pH <6) are


(1a)

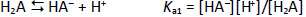





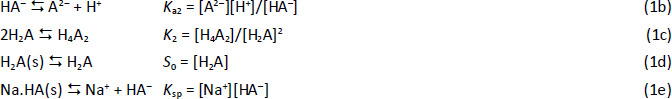



where *S*_0_ is the intrinsic solubility of the uncharged (monomeric free acid) form of the molecule. Based on the above relations, the total concentration of the weak acid in the saturated aqueous phase, *A*_tot_^aq^, is the total solubility, *S*_T_, which can be expressed solely in terms of equilibrium constants and pH:



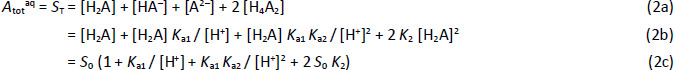



In logarithmic form, base 10,


(3)

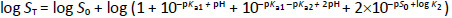



In the thermodynamically valid constant ionic medium (CIM) activity scale [17], the reference ionic strength may be set to *I*_ref_ *>* 0. In such a framework, all equilibrium constants are expressed in terms of concentrations. In the multiple-pH solubility data analysis, all constants are adjusted for local deviations from *I*_ref_, using the Stokes-Robinson hydration equation [16,17]. In the case of uncharged species, similar adjustments for local deviations from *I_ref_* are made using the salting out equation approach described below.

In the CIM framework, the operational p_a_H scale (pH meter readings) is standardized to the concentration scale, p_c_H, using the four-parameter equation [27]


(4)

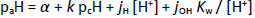



where *K*_w_ is the ionization constant of water [28]. The *j*_H_ term corrects p_a_H readings for the nonlinear pH electrode response due to liquid junction and asymmetry potentials in highly acidic solutions (pH <2), while the *j*_OH_ term corrects for high-pH nonlinear effects [17]. Since Schill standardized the electrode to read on the concentration scale and since all saturation data were for pH <6, it was assumed here that *α* = *j*_OH_ = 0 and *k* = 1. The *j_H_* was adjusted during the regression analysis since many pH values were <1 in the 1 M NaCl set (Fig. 2).

In the analysis of the Schill data, p*K*_a2_ was selected as 7.12. (Since most of the data are in the acidic range, p*K*_a2_ is of minor role here.) The rest of the constants in Eq. (3) were determined by nonlinear regression analysis (*i.e*. mass action model), the details of which are described elsewhere [16,17]. The data were first evaluated as Schill had done, and essentially the same constants were determined as those listed in Figure 2. For those constants, self-interaction (aggregation) was not included (as per Schill’s assumption).

### Salting activity model for uncharged species

The Setschenow equation [1,2,10-15] describes the salting effect on nonelectrolyte (*i.e*. H_2_A) *total* aqueous solubility, *S*_T_, as


(5)

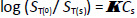



where 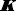 is the empirical Setschenow coefficient (M^-1^) and *C_s_* is the concentration (M) of the added salt. S_T(0)_ and S_T(s)_ are the neutral solute *total* solubility values in pure water and in water containing salt, respectively. 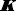 is positive in the case of salting-out and negative in the case of salting-in processes. Generally, the equilibrium constants in Eq. (5) can represent a solute partitioning process between different phases.

If self-interaction (aggregation) is hypothesized, then a modified form of the Setschenow equation may need to be invoked [2,4,11]:


(6)

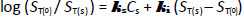



where 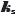 and 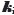 are nonelectrolyte salting and self-interaction parameters, respectively. Only for low *S*_T_ or in the absence of nonelectrolyte self-interaction will the empirical Setschenow constant, 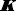, be equal to the salting parameter, 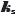.

Usually 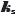 is determined from oil-water partition data using small volumes of water-immiscible oil, where aggregates would not be expected to enter the oil phase [11]. Eq. (5), cast in the form of partition coefficients, may be used to determine 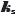. The concentration of the monomer is measured in the oil phase and the corresponding value in the water phase is calculated from mass balance to determine the partition coefficient. Once 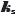 is known, then 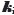 can be calculated form Eq. (6). This is the approach used by Al-Maaieh and Flanagan [11] to determine the self-interaction parameter for caffeine, theophylline, and theobromine (*e.g*., 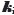 = -2.06 M^-1^ for caffeine in 1 M Na_2_SO_4_ solution, indicating less aggregation with increasing salt concentration [11]).

A different approach was used presently: *k_s_* was determined by substituting into Eq. (5) the intrinsic (monomer) solubility, *S*_0_ (*cf*. Eq. (1d)), determined by regression analysis. In Eq. (5), 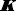 was taken to be 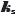; *S*_T(0)_ and *S*_T(s)_ were substituted with *S*_0(0) *_and *S*_0(s)_, respectively. Once 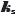 was so determined, then 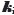 was calculated from Eq. (6).

### Abraham linear free energy descriptors used to predict the salting-out constant of BTB

Abraham’s five linear free energy solvation descriptors (*A, B, S_π_, E*, *V*) have been used to describe the distribution of nonelectrolytes between two phases [29,30]. *A* is the H-bond acidity and *B* is the H-bond basicity of the solute. *S*_π_ is the dipolarity/polarizability, *E* is an excess molar refractivity in units of (cm^3^/mol)/10, and *V* is the McGowan characteristic molar volume in units of (cm^3^/mol)/100.

Endo *et al*. [4] used the Abraham model to predict salting-out constants (M^-1^):


(7)

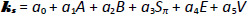



The *a*_0_-*a*_5_ coefficients in Eq. (7) were determined by multi-linear regression (MLR): 0.112, -0.047, -0.060, -0.042, -0.020, and 0.171, respectively [4]. Evidently, large molecules increase the salting-out behavior, and polar molecules act in the opposite direction.

Presently, the a-coefficients were re-determined by partial least squares (PLS) regression (open-source package from https://cran.r-project.org/web/packages/pls), using 142 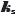 values as the training set (136 values compiled/measured by Endo *et al.* [4] and six derived here from the data of Furia *et al.* [12]) to predict the *k_s_* value of bromothymol blue. Values of the Abraham descriptors for bromothymol blue were calculated from its 2D structure using the ABSOLV algorithm [30] (*cf*. www.acdlabs.com).

## Results and discussion of data re-analysis

The nature of the structure of the BTB free acid species in saturated solutions is still controversial and has been discussed for many decades. Several different monomeric structures for the H_2_A species may exist/co-exist (two zwitterion tautomers and a closed sultone ring uncharged form) in saturated solutions below pH 3, each with its own associated ‘micro-constant’ for p*K*_a1_. An electrostatically-bound dimer based on two zwitterions oriented with opposite-charge groups at contact distances may be feasible. Other structures are possible for a BTB dimer. A further complication is that the structural form of the BTB free acid in the crystal lattice may undergo tautomeric re-arrangement upon dissolution, posing a challenge to the canonical definition of intrinsic solubility, since the form of the substance would be different between the solid state and the solution phases. Definitive support for structures of BTB species in saturated aqueous solutions is sketchy and is mostly inferred from spectrophotometric measurements in sub-saturated solutions.

The speciation analysis of solubility-pH data is based on thermodynamic principles, formulated in terms of ‘macro-constants.’ Structural questions can only be addressed *indirectly*. In this communication, three different model strategies were hypothesized and tested against Schill’s solubility-pH data.

### MODEL A. Dimer-free, single pK_a1_ model, based on an unusually-high salting-out factor

Our first strategy was to be open to the possibility of an unusually high 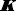, to assume that (i) dimers did not form in the pH <3 region, and (ii) one of Schill’s p*K*_a_ values was more reliable (but both values could not be simultaneously ‘correct’). We selected (and refined) p*K*_a1_ = 1.43 ±0.09 (0.1M NaCl) since its value would be least affected by the salting-out effect of bromothymol blue. A fit of the two apparent intrinsic solubility values to *C*_s_ (Eq. (5)), yielded intercept salt-free value of p*S*_0_ = 5.10 and the slope factor 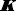 = 0.778. The refinement of the model in the case of 0.1 M NaCl (keeping p*K*_a1_ fixed at 1.43) yielded at *I*_ref_ = 0.1 M: p*S*_0_ = 5.17±0.02 (using the high 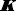 to correct for the activity of the net zero charge H_2_A species) and p*K*_sp_ = 4.41±0.03 (goodness-of-fit, GOF = 0.50).

However, when the above model was applied to the 1.0 M NaCl case, it was not feasible to adhere to the above assumption (i) at the high salt level. It was not possible to fit the Schill data without invoking the mixed-charge dimer, H_2_A.HA^–^, since such a species would account for the apparently lower p*K*_a1_ in 1 M NaCl solutions reported by Schill, while maintaining the slope in the 1-3 pH region at +1 [17]. Also, it was necessary to invoke *two* salt species, with the new addition being Na.H_2_A.HA(s), needed to explain the data in the pH 2.5-3.5 region. In the refinement of the data, p*K*_a1_ and p*S*_0_ were kept fixed at 1.43 and 5.17 (since the 0.1 M NaCl data defined these values). The refined constants, also with reference to *I*_ref_ = 0.1 M, were log *K*_H3A2_ = 5.87±0.03, p*K*_ps (NaH3A2)_ = 4.93 ±0.02, and p*K*_sp(NaHA)_ = 4.33 ±0.02 (GOF = 0.35) for the case of 1.0 M NaCl.

### MODEL B. Fitting a single pK_a1_ to Schill’s data, assuming a neutral dimer forms

In our next strategy, it was hypothesized that there can only be a single p*K*_a1_ for bromothymol blue and its value would not likely be either 1.48 or 1.00, as Schill had reported. We hypothesized that a neutral dimer formed. It was reasoned that this caused the *S_T_* near pH p0 to be different in the 0.1 and 1.0 M NaCl solutions. This was hypothesized to be the explanation for the two values of the reported *pK_a1_*. Since there appeared to be less self-association in the 1 M NaCl solutions, the new value for the *pK_a1_* was sought in that medium, with *I*_ref_ was set to 1.0 M. Starting with the Schill p*K*_a1_ = 1.00 as a fixed constant, the p*S*_0_, log *K*_2_, and p*K*_sp_ constants were determined iteratively by nonlinear regression. Next, these three refined values were fixed, and p*K*_a1_ was subjected to regression. Its value decreased steadily. It was then fixed, and the process was repeated with the first three constants. After several cycles of this ‘block-diagonalization’ refinement, the minimum errors in all four constants were reached, and the overall goodness-of-fit (GOF) [17] settled at its minimum value. The excessive correlations between the p*K*_a1_ and the other three constants did not permit their simultaneous determination, so the ‘block-diagonalization’ approach was used. The p*K*_a1_ so determined (+0.217) was then applied to the 0.1 M NaCl set. The same iterative process led to convergence.

It soon became evident that a range of reasonable p*K*_a1_ values could be proposed and the fitting of the Schill data (Fig. 2) would be equally good (as indicated by the minimum GOF reached). Since the correlation between p*K*_a1_ and the other constants was extreme, the ‘block-diagonalization’ minimum lay over a very shallow well, and normal experimental errors in solubility measurement caused havoc to pin down the ‘best’ p*K*_a1_ value.

### MODEL C. Applying an independently-measured single pK_a1_ to Schill’s data, assuming a neutral dimer forms

Attention was then directed to finding an independently determined value of *pK_a1_* for the further analysis of the Schill solubility data. The only value found was reported by Gupta and Cadwallader [15]: p*K*_a1_ = -0.66. The authors collected UV-visible spectroscopic data in the pH interval from 0.92 to -1.08 (concentration scale), where the pH of distilled water was adjusted using 12 M HCl. At -log [H^+^] = -0.662, the concentration of the anion was found to be equal to the concentration of the uncharged species. This defines the value of the p*K*_a1_ at ionic strength 10^+0.662^ = 4.59 M. The Stokes-Robinson hydration equation was then used to harmonize this value to the CIM activity scale (*I*_ref_ = 1.0 M), to obtain p*K*_a1_ = -1.18, the value ultimately to be used as a fixed contribution in the regression analysis of the Schiff solubility data.

### Model selection

Model A described above, with the single p*K*_a1_ = 1.43 (although it could not support a dimer-free case for both 0.1 and 1.0 M NaCl media), is consistent with the ‘red’ species in Figure 1 as shedding a proton from a protonated carbonyl group in the cyclohexadienone zwitterion. Also, Model A supports a more soluble ‘red’ zwitterion with p*S*_0_ = 5.17, compared to 8.02 in Model C (Table 1). However, p*K*_a1_, derived from Schill’s data, depends on two assumptions that could not be fully supported since it was necessary to invoke the presence of a mixed dimer, H_2_A.HA^–^, as well as a salt based on the dimer, in 1.0 M NaCl solutions. The latter two species could not be fit to the data in 0.1 M solutions. Model A was also dependent on an unusually high value of the empirical constant, 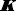 = 0.778.

Model B can be dismissed since a specific value of p*K_a_*_1_ could not be determined definitively from the solubility data by the ‘block diagonalization’ refinement procedure.

Model C is attractive since the p*K*_a1_ value was derived from an independent study using UV/Vis data taken in sub-saturated solutions [15]. The so-determined value, adjusted to *I*_ref_ = 1.0 M is -1.18, a much lower value than either of the two apparent values reported by Schill. It may represent the shedding of the proton from a protonated sulfonic acid group in an open sultone ring form of BTB, possibly present in the sub-saturated solution used by Gupta and Cadwallader [15]. However, structural issues cannot be directly addressed by the thermodynamic speciation analysis presented here.

It was decided in this communication to focus further discussion on Model C. Also, the Setschenow constant based on Model C is much more in line with values taken from the literature for many molecules.

### Mass action equilibrium model

**Figure 3.**
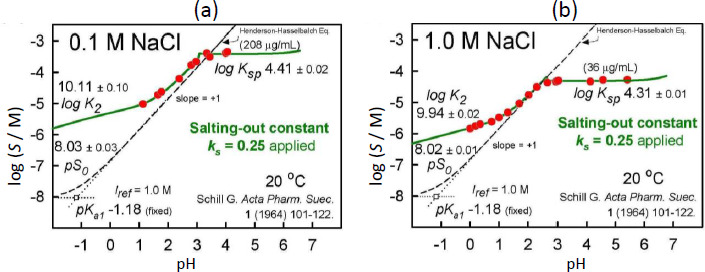
Summary of the re-analysis of bromothymol blue solubility-pH data reported by Schill [14], incorporating the p*K*_a1_ reported by Gupta and Cadwallader [15] (adjusted to *I*_ref_ used here). The points in the green solid curves were calculated after correcting for salting-out effects of the uncharged saturated species. The curves decrease with pH <0 since salt concentrations are elevated due to addition of titrant.

The results of the final re-analysis of Schill’s two sets of saturation solubility-pH data (in 0.1 and 1.0 M NaCl media), employing the Gupta-Cadwallader [15] p*K*_a1_ (transformed to the CIM activity scale), are listed in Table 1 and depicted in Figure 3.

### Dependence of equilibrium constants on ıonic strength (Stokes-Robinson hydration equation corrections)

When the ionic strength for a given point in a log *S-*pH titration is calculated to stray from the designated *I*_ref_ value (1.0 M here), it is beneficial to adjust the equilibrium constants in Eqs. (1a), (1b), and (1e). The Debye-Hückel equation is often used for this adjustment. However, the latter equation becomes less accurate as ionic strength exceeds about 0.3 M [17]. Since the Gupta-Cadwallader [15] p*K*_a1_ was measured at *I* = 4.59 M, it was not expected that the simple Debye-Hückel equation would be suitable in correcting for activity changes.

**Figure 4.**
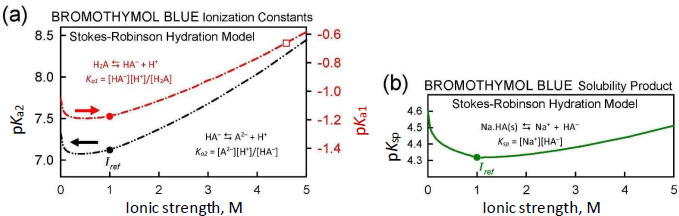
**(a)** Dependence of p*K*_a1_ and p*K*_a2_ on ionic strength, according to the Stokes-Robinson hydration model. **(b)** Dependence of the negative logarithm of the solubility product, *K*_sp_ = [Na^+^][HA^–^] on ionic strength.

A more comprehensive correction scheme, which may still be useful for *I* < 5 M, involves the application of the Stokes-Robinson hydration theory [16,17]. The scheme is coded into *p*DISOL-X. Figure 4 shows how the ionization constants and the solubility product depend on ionic strength in the Stokes-Robinson model. The Gupta-Cadwallader reported p*K*_a1_ = -0.66 (unfilled square in Fig. 4a) was adjusted to -1.18 at *I*_ref_ = 1.0 M (solid red circle in the upper curve in Fig. 4a). The solubility product, *K*_sp_ (*cf.*, Eq. (1e) and Fig. 4b), has a less steep ionic strength dependence, compared to those of p*K*_a1_ and p*K*_a2_.

Since ionic strength is not defined for uncharged species, the Stokes-Robinson scheme is not directly applicable to the intrinsic solubility (Eq. (1c)) and dimerization of uncharged species (Eq. (1d)) equilibrium constants.

However, the next section addresses how salt levels can affect the activity of uncharged species.

#### Salting model

**Figure 5.**
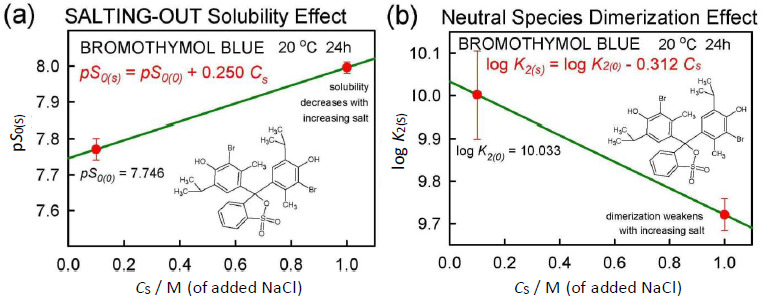
**(a)** Salting-out and **(b)** dimerization effects in bromothymol blue saturated solutions, as a function of the amount of added salt, NaCl. These are the apparent refined constants when 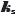 was set to zero. Two salt points preclude the possibility to test nonlinear relationships, as seen in cases of substituted phenolic acids covering a wide range of salt concentrations [12].

The application of Eq. (5) to the *total* solubility values in Figure 2 resulted in the linear relationship, p*S*_T(s)_ = 5.10 + 0.778 *C*_s_, where the slope factor is 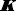 = 0.778. Figure 5a shows the equivalent of Eq. (5) based on intrinsic (monomer) solubility values. The slope factor listed in the figure corresponds to the true salting-out parameter, 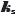 = 0.250. When this salting-out value is substituted into Eq. (6) for the case of *C*_s_ = 1.0 M,


(7)

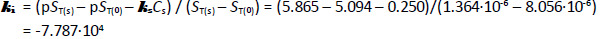



Its magnitude exceeds that of any other reported 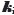 values, as far as we could find.

Once 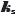 and 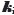 were determined, Eq. (6) can be used to calculate the intrinsic and dimerization equilibrium constants of BTB over a range of salt concentrations. This is evident in the green solid curves in Figure 3 for pH values below zero, where titrant additions to achieve very low pH increase the salt content in the saturated solutions.

### Distribution of aqueous-phase species in saturated solutions of bromothymol blue

**Figure 6.**
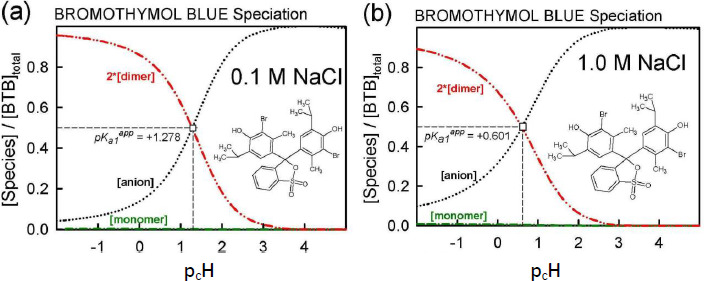
Distribution of species in **(a)** 0.1 M NaCl and **(b)** 1.0 M NaCl containing solutions, based on the constants in Table 1; p*K*_a1_ = -1.18 (*I*_ref_ = 1.0 M).

The equilibrium model developed using Schill’s solubility-pH data and Gupta-Cadwallader p*K*_a1_ (Table 1) allows for the calculation of the distribution of species at the two levels of salts considered, as illustrated in Figure 6. It is evident that the monomer concentration (green dash-dot curve in Fig. 6) is dwarfed by that of the dimer (red dash-dot-dot curve in Fig. 6).

In the 0.1 M NaCl medium at pH 1.28 (Fig. 6a), 50% of the total bromothymol blue is in the monoanionic form and half is essentially in the dimeric form. So, in Figure 2, the Schill-proposed ionization constant, 1.48, appears to be indicating the equilibrium between anionic HA^–^ and the dimeric (H_2_A)_2_ species (and not the monomer). Similarly, in the 1.0 M NaCl medium at pH 0.60 (Fig. 6b), 50 % of BTB is in the monoanionic form and half is essentially in the dimeric form. The Schill-proposed ionization constant, 1.00, again appears to be indicating the equilibrium between anionic HA^–^ and the dimeric (H_2_A)_2_ species.

At the half-point pH_1/2_, [HA^–^] ≈ [H_4_A_2_]. From Eq. (2c), the estimate of p*K*_a1_ ≈ -log 2 + p_c_H + p*S*_0_ – log *K*_2_. For the 0.1 M and 1.0 M NaCl cases, the resulting approximate p*K*_a1_ values are -1.3 and -1.4, respectively, compared to the -1.18 used (Fig. 3). The slight differences may indicate the shortcomings in attempts to standardize the pH electrode in such low pH regions, as per Eq. (4), given the uncertainty over junction potentials and the uncertainty in adjusting the p*K*_a1_ = -0.66 (at 4.59 M ionic strength) from Gupta and Cadwallader [15] to the selected reference ionic strength of 1.0 M.

### Comparison of bromothymol blue (BTB) to thymol blue (TB) equilibrium speciation

The ‘Flexible-Acceptor’ GSE consensus model [31] predicts the intrinsic solubility values of thymol blue (TB) and BTB as 6.67 and 7.68, respectively. Given the similarity of structures, it might be anticipated that TB and BTB would possess similar equilibrium reactions. Shimada *et al.* [32] analyzed the spectral changes (300 to 650 nm) of TB solutions in the pH -0.02 to 4.35 interval. Solutions of TB gradually change from red to yellow as pH is raised in the interval studied. One prominent and two minor isosbestic points were evident in the spectra. Using principal components analysis (PCA) and alternative least-square (ALS) regression, the authors concluded that two major species accounted for the TB equilibria in the strong acid solutions. The apparent p*K*_a1_ of TB was determined to be 1.54 in 0.1 M and 1.45 at 1.0 M ionic strength solutions [32]. The red species was most plausibly the zwitterionic form of TB, possibly a carbocation or its resonance equivalent, like the left structure in Figure 1 of BTB. The colorless uncharged sultone closed ring form was not expected to be stable in the polar medium of water (although it is present in the crystal structure reported by Yamaguchi *et al.* [33]). Since the bromine atoms in BTB are electron withdrawing substituents, the p*K*_a1_ value of BTB was suggested (without reference) to be -1.3 (compared to +1.6 of BT), and the red form was anticipated to be dominant in highly acidic aqueous medium [32].

From the rigorous analysis of spectrophotometric, potentiometric, and conductimetric data at 25 °C of TB in 1.1 M ionic strength (NaCl) aqueous solutions, Balderas-Hernández *et al*. [34] reported p*K*_a2_ = 8.90 and the constants for the reactions


(8)

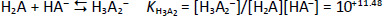




(9)

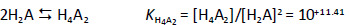



The above two constants were originally reported on the cumulative ‘stability’ basis. The conversions to the step-wise basis [17] in Eqs. (8) and (9) employed p*K*_a1_ = 1.45 for TB from Shimada *et al.* [32]. The latter constant taken with those from Eqs. (8) and (9) suggest that the proton dissociation constant of the H_4_A_2_ dimeric species has a dimeric ionization constant of 1.38, as indicated in Eq. (10).


(10)

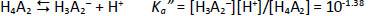



Hence, the anionic dimer in TB is expected to be dominant above pH 1.38, while the zero net-charge dimer is expected to prevail below pH 1.38. Attempts to incorporate the above model into the Schill data for BTB were not successful. Given the BTB p*K*_a1_ = -1.18, Schill’s solubility-pH data did not support the inclusion of the H_3_A_2_^–^ species in both the 0.1 and 1.0 M NaCl solutions, as discussed earlier in the context of Model A.

It is quite remarkable that the uncharged dimeric species from the Balderas-Hernández *et al*. [34] TB study is so close in value to that reported here for the BTB. The predicted TB intrinsic solubility is an order of magnitude higher than that of BTB. It is reasonable to view the TB and BTB system as being similar, with ionizations shifted to lower values of pH in the case of BTB because of the bromine substituents.

### Prediction of k_s_ using the Abraham model and literature values of salting-out constants

The 142 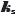 values mentioned earlier (including the provided Abraham descriptors) [4,12] were used to re-determine the Abraham coefficients in Eq. (7) by PLS regression. The a_0_-a_5_ coefficients were determined as 0.090, -0.073, -0.064, -0.039, -0.002, +0.188, respectively. The regression coefficients are comparable to those reported by Endo *et al.* [4]. Highly-polar molecules are predicted to have weak salting-out effects (a_1_-a_4_ factors). On the other hand, large molecules are expected to be highly sensitive to salting-out. The ABSOLV-calculated values of the Abraham descriptors for bromothymol blue (left-most structure in Fig. 1) are *A* = 0.33, *B* = 1.28, *S_π_* = 2.48, *E* = 2.94, and *V* = 3.99. The polar terms contribute 21 % (negative) to the predicted effect and the volume term contributes 75 % (positive). Using the coefficients reported by Endo *et al*. [4], predicted ***k***_s_ = 0.54 for BTB. Using the PLS-derived coefficients here, the predicted 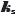 = 0.63. The two predictions are substantially higher than the 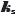 = 0.25 value determined from the data of Schill [14] using Model C. It may be that the training set of 142 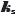 values did not cover the chemical space of molecules like bromothymol blue or molecules that form very stable water-soluble oligomers.

### Limitation of the bromothymol blue equilibrium model

As discussed elsewhere [9, 17-23], it is not possible to determine the exact degree of aggregation of *neutral* species solely from the solubility*-*pH data (Case 1a in Fig. 6.6 in ref. [17]). It is necessary to use other stoichiometry-sensitive methods, such as ESI-Q-TOF-MS/MS [25]. The simplest stoichiometry (dimer) was modeled here, but the actual species may be a tetramer, a higher-order/mixture of (H_2_A)_n_ oligomers [14,15]. Any of such species can fit the solubility data equally well for low sample concentrations. The pH_1/2_ values in Figure 6 would remain unchanged for low sample concentrations.

The p*K*_a1_ value is not easy to measure independently since its value is negative, and aggregation is a confounding complication. Standardization of the pH electrode is likely to be a challenge. The measurement of the ionization constant in cosolvent mixtures (*e.g.*, methanol-water) is expected to increase the p*K*_a1_ value, perhaps making the determination more reliable. Potentiometric titrations, which can simultaneously determine both the solubility and the p*K*_a_ can be effective [17, 35].

Additional measurements of the saturation solubility of bromothymol blue for pH >6 might possibly support some of the suggestions proposed by Schill regarding the nature of self-association he studied by UV-vis spectrophotometry in sub-saturated solutions.

## Conclusion

Bromothymol blue reveals complex speciation chemistry in saturated aqueous solutions, which is influenced strongly by the presence of high concentrations of NaCl. Schill’s [14] extensive saturation solubility-pH measurements (pH from 0 to 5 in 0.1 and 1.0 M NaCl solutions) of the practically-insoluble diprotic weak acid, bromothymol blue (6 ng/mL monomer solubility), was re-analyzed and harmonized using a general mass action approach. A ‘normal’ salting-out constant, 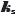 = +0.25 M^-1^ was derived. The formation of self-aggregates (here treated as dimers, with zero-salt *log K*_2_ = 10.0 ± 0.1 M^-1^) in strongly acidic solutions raised the *total* solubility of bromothymol blue nearly 500-fold to zero-salt log *S*_T_ = -5.10. The data analysis in the present study critically depended on the p*K*_a1_ = -0.66 (at 4.59 M ionic strength) reported by Gupta and Cadwallader [15]. It was possible to estimate the self-interaction constant, 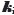 = -7.787·10^+4^ M^-1^, an unusually high value, reflecting the tendency of bromothymol blue to form oligomers in strongly acidic solutions.

## Figures and Tables

**Table 1.** Refined constants - bromothymol blue, 20 °C, 0.1 and 1.0 M NaCl Media (*I*_ref_ = 1.0 M)

[NaCl] / M	p*K*_a1_	p*S*_0_	log *K*_2_	p*K*_sp_	*I_avg_* / M^*a*^	*n* ^b^	*GOF^c^*
0.10	-1.18 ^d^	8.03 ±0.03	10.11 ±0.10	4.41 ±0.02	0.11	10	0.48
1.00	-1.18 ^d^	8.02 ±0.01	9.94 ±0.02	4.31 ±0.01	1.07	15	0.18

^a^Average ionic strength. To minimize dilution effects, 12 M HCl titrant was used in the refinement model.

^b^Number of pH points.

^c^*GOF* = goodness-of-fit [16], based on assigned individual *log S* errors of 0.1 log unit.

^d^p*K*_a1_ = -0.66 at *I* = 4.59 M from Gupta and Cadwallader [15] was transformed to *I*_ref_ = 1.0 M here, using the Stokes-Robinson hydration equation.
